# Changes in K^+^ Concentration as a Signaling Mechanism in the Apicomplexa Parasites *Plasmodium* and *Toxoplasma*

**DOI:** 10.3390/ijms24087276

**Published:** 2023-04-14

**Authors:** Benedito M. Dos Santos, Jude M. Przyborski, Célia R. S. Garcia

**Affiliations:** 1 Department of Clinical and Toxicological Analysis, School of Pharmaceutical Sciences, University of Sao Paulo, Sao Paulo 05508-000, Brazil; 2 Department of Biochemistry and Molecular Biology, Interdisciplinary Research Center, Justus-Liebig University, 35390 Gießen, Germany

**Keywords:** calcium, GPCR, malaria, PfSR25, *Plasmodium berghei*, *Plasmodium falciparum*, potassium, *Toxoplasma gondii*

## Abstract

During their life cycle, apicomplexan parasites pass through different microenvironments and encounter a range of ion concentrations. The discovery that the GPCR-like SR25 in *Plasmodium falciparum* is activated by a shift in potassium concentration indicates that the parasite can take advantage of its development by sensing different ionic concentrations in the external milieu. This pathway involves the activation of phospholipase C and an increase in cytosolic calcium. In the present report, we summarize the information available in the literature regarding the role of potassium ions during parasite development. A deeper understanding of the mechanisms that allow the parasite to cope with ionic potassium changes contributes to our knowledge about the cell cycle of *Plasmodium* spp.

## 1. Introduction

The parasite that causes malaria (*Plasmodium* genus) is a unicellular eukaryote belonging to the phylum Apicomplexa and the family Plasmodiidae. It has a complex biological cycle consisting of different development stages comprising two phases: (i) asexual, where the parasites are found in the vertebrate host; and (ii) sexual, which occurs in the anopheline invertebrate host. During its biological cycle, the parasite undergoes morphological and biochemical changes, which make it possible to distinguish between the different stages [1,2].

Transmission occurs as a by-product of feeding, with infected female anopheline mosquitoes injecting sporozoites into the dermis of the vertebrate host, from where they eventually enter the bloodstream. Sporozoites in the bloodstream migrate to the liver and invade liver cells (hepatocytes), where they multiply asexually; this asymptomatic period lasts, on average, between six and fifteen days. At the end of the hepatic phase, the merozoites are released by rupture of the hepatocytes and subsequent invasion of erythrocytes. The erythrocytic cycle consists of parasites passing through the ring, trophozoite, and schizont stages [3,4,5,6,7].

Throughout its life cycle in the vertebrate host, the malaria parasite is exposed to extreme environmental changes, including the host’s immunological defenses, temperature, and ion concentrations, as it moves between microenvironments. Ions were shown to play an essential role in several biological processes of parasite development [8]. Regarding the concentration of potassium ions (K^+^), the parasite is exposed to a variation of 5 mM when outside the blood cells to 140 mM inside the parasitized cell [9]. Several studies have demonstrated the importance of the K^+^ ion as a regulator of events during the development of various Apicomplexan parasites, including the malaria parasite. In this present contribution, we summarize the primary information available in the literature about the essential role of K^+^ in the infectivity, development, and survival of *Plasmodium* spp.

## 2. Differences in Ion Concentrations in the Microenvironment Activate Signaling Pathways Required for Infection

After the mosquito injects sporozoites into the vertebrate host, infection of the hepatocytes is the next step in the parasite’s development. When in contact with the intracellular environment of these cells, the parasite initially migrates without forming a parasitophorous vacuole until it eventually reaches a cell in which it develops [5]. During this process, the parasite moves from the intracellular to the extracellular environments, and it is exposed to different [K^+^], from [5 mM] in the extracellular environment to [140 mM] when the parasite resides inside the host cells [10]. By studying the process of sporozoite motility to and through liver cells, Mota and coworkers suggested that this process of motility and cell-crossing is essential for the parasite to activate the signaling pathways necessary for its subsequent development in the hepatocytes. Incubation of sporozoites with host cell extracts increases the secretion of proteins involved in the invasion. Thus, parasite migration through cells induces exocytosis of apical organelles required for infection and represents an essential step in the parasite lifecycle [5,11].

Using murine malaria parasites (*Plasmodium berghei*), Kumar et al. (2007) observed that sporozoites incubated in a high [K^+^] buffer (142 mM) and subsequently placed in liver cell culture (HepG2) showed an increase in infectivity and reduced cell passage activity compared to sporozoites incubated in Na^+^-rich buffer [10]. When similar experiments were carried out in the presence of K^+^ channel blockers (20 mM tetraethylammonium chloride and 0.5 mM quinine), there was a decrease in infectivity compared to sporozoites treated with K^+^-rich buffer [10]. We interpret these data to suggest that: (i) the detection of K^+^ ions by the parasite inside cells and in the extracellular environment regulates cell infectivity, and thus, it is necessary for the progression of the parasite life cycle; (ii) the passage of sporozoites through hepatocytes may trigger the activation of signaling pathways induced by migration through host cells [5,10,11,12,13].

In *T. gondii*, Moudy et al. (2001) evaluated the ionic environment in the parasite egress process by investigating the influence of [Ca^2+^], [Na^+^], [Cl^−^], and [K^+^] from intracellular to extracellular levels in HFF cells. The authors showed that the only ion that plays a role in this process is K^+^; when [K^+^] is similar to cytoplasmic levels, the egress does not occur, but under low [K^+^] or total absence of this ion, the exit is more efficient [14]. Secretion of the microneme is critical for the successful infection of new cells and the progression of the life cycle. The environment in which the parasites find themselves has been shown to correlate directly with the activation of this process, which requires Ca^2+^ as an intracellular second messenger [15,16,17]. An increase in Ca^2+^ concentration is associated with significant changes in parasite morphology, in addition to promoting the secretion of proteins involved in invasion and egress from the host cell [18]. An increase in Ca^2+^cyt initiates a Ca^2+^ signaling cascade that promotes parasite motility and egress from the cell. Recent findings have shown that intracellular *T. gondii* tachyzoites are able to capture Ca^2+^ from the host cytoplasm to reach a cytosolic Ca^2+^ threshold required for successful egress; furthermore, this exit process is favored by decreasing [K^+^], indicating that the reduction of [K^+^] around the parasite is one of the factors involved in the egress rate [19].

Undoubtedly, the activation of the parasite’s motility machinery is a crucial point for the successful infection of new cells; behind this process, Ca^2+^ acts as an irreplaceable messenger transmitting to the intracellular medium the external stimuli from the environment [20,21]. Similar to *Plasmodium*, *Toxoplasma* also benefits from information coming from the extracellular environment to complete its life cycle, and the detection of [K^+^] is one of these mechanisms. One of the pieces in the K^+^ signaling in T. gondii is the apical protein Guanylate Cyclase (TgGC). Conditional knockdown of TgGC presents parasites defective in motility, adhesion, invasion, and egress of the host cell and is a critical protein for capturing changes in external pH and extracellular [K^+^] to activate the Ca^2+^cyt increase [22]. From this point, we draw attention to K^+^, an ion that is present in different concentrations in the microenvironments that parasites pass through during their development.

## 3. K^+^ in the Invasion of Erythrocytes and Progression of the Erythrocytic Cycle

After the hepatocyte cycle, the parasite reaches the bloodstream, beginning its intraerythrocytic cycle, where they reproduce asexually within the erythrocytes of its vertebrate host. Studies from the Kirk group indicate that considerable changes in cell membrane permeability occur in erythrocytes infected by *P. falciparum* ±15 h after the invasion and that these are detrimental to the normal ion balance. The loss of K^+^ and Na^+^ across the membrane increases, and the activity of the Na^+^/K^+^ pump decreases; these processes result in the replacement of K^+^ ions by Na^+^ ions in the erythrocyte cytosol [23,24,25,26,27]. The Na^+^ concentration in the cytosol of the infected cell is high, while the parasite maintains a low cytosolic Na^+^ concentration [27,28].

In 2016, Spillman reported a mechanism by which, via a Na^+^-ATPase from *P. falciparum*, PfATP4 expels Na^+^ from its cytosol into that of the host cell. In addition, the authors described PfATP4 as a target for spiroindolones, as spiroindolones disrupt the Na^+^ homeostasis of the parasite. Furthermore, it has been shown that mutations in PfATP4 confer resistance to antimalarial spiroindolones [29]. In another study by the group, Winterberg and Kirk (2016) reported a correlation between the intracellular concentrations of Na^+^ and K^+^ in red blood cells infected with *P. falciparum*. Using a high-sensitivity HPLC assay to measure intracellular Na^+^ and K^+^, the authors showed that a new spiroindolone antimalarial candidate, KAE609, can promote the disruption of parasite ion homeostasis by increasing [Na^+^] and decreasing [K^+^] after 30 min of 50 nM treatment [30].

Proteins on the surface of merozoites and proteins secreted by apical membrane-associated organelles, rhoptries, and micronemes actively participate in the erythrocyte invasion process. In 2010, Singh and colleagues found that exposure of merozoites to low [K^+^] (5 mM), as seen in blood plasma, promotes a PLC-dependent increase in cytosolic calcium levels (Ca^2+^cyt) [31]. This increase in Ca^2+^cyt would be associated with the release of microneme proteins, such as erythrocyte-binding antigen (EBA175) and apical membrane antigen-1 (AMA-1), to the merozoite surface [26]. The interaction of EBA175 with its receptor on erythrocytes, glycophorin A (glyA), restores basal Ca^2+^cyt levels and triggers the release of rhoptry proteins [31]. The authors identify for the first time an external signal responsible for the sequential release of micronemes and rhoptries proteins during erythrocyte invasion, this signal being the changes in [K^+^] in the parasite’s microenvironment.

In a very elegant study from the Chitins’ lab, the authors treated *P. falciparum* merozoites, isolated in [140 mM K^+^], with the calcineurin inhibitors FK506 and cyclosporin A, before transferring the parasites to an environment with low K^+^ concentration (5 mM). The shift of exposure of merozoites to [K^+^] from 140 to 5 mM triggered the release of microneme proteins, mainly the apical membrane antigen 1 (AMA1) protein. The use of calcineurin inhibitors resulted in the inhibition of AMA1 secretion to the merozoite membrane, preventing the process of erythrocytes invasion by the parasite [32,33]. This seminal work allows the authors to propose the involvement of a [K^+^]-dependent and calcium pathway in the erythrocyte invasion process.

## 4. *Plasmodium falciparum* Serpentine Receptor 25 (PfSR25), a GPCR-like Receptor Responsible for the Transmission of the K^+^ Stimulus to the Interior of the Parasite

**Figure 1:**
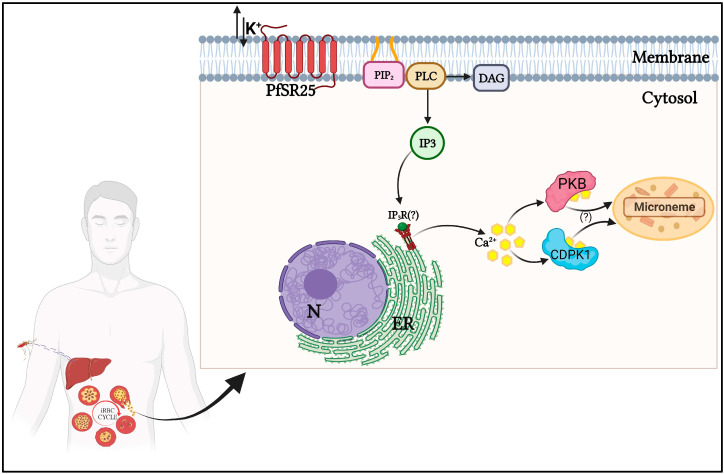
Schematic representation of the signaling pathway involving K^+^ in *Plasmodium* spp. during its asexual developmental phases. In the erythrocytic stage, the exposure of *P. falciparum* to variations in [K^+^] is perceived by the GPCR-like PfSR25, which transmits the signal through the activation of phospholipase C (PLC). After PLC activation, inositol 1,4,5-triphosphate (IP_3_) and diacylglycerol (DAG) are formed. IP_3_ activates an IP_3_ receptor (hypothetical IP_3_R) to release calcium (Ca^2+^) from the endoplasmic reticulum (ER). The increase in cytosolic levels of Ca^2+^ activates the Ca^2+^-dependent protein kinases CDPK1 and PKB, leading to the release of microneme proteins essential for the infection of new erythrocytes. Created with BioRender.com.

The complex signaling pathways present in *P. falciparum* led Madeira et al. (2008) to search for G protein-coupled receptors (GPCRs) in the *P. falciparum* genome. Through a robust bioinformatic analysis of the parasite genome, the authors identified four candidates for GPCR receptors, namely, PfSR1, PfSR10, PfSR12, and PfSR25 [34]. In 2017, the *P. falciparum* serpentine receptor 25 (PfSR25) was identified as responsible for sensing the [K^+^] shift stimulus [35] (Figure 1).

In the work of Moraes and colleagues (2017), the authors generated a knockout of the GPCR candidate PfSR25 (PfSR25^−^). This tool showed that this serpentine receptor acts to sense the stimuli resulting from [K^+^] changes in the microenvironment, in which the parasite travels during its erythrocytic cycle. The authors investigated whether SR25 could modulate the calcium signaling in parasites at mature trophozoite stages isolated from red blood cells and incubated with high K^+^/low Na^+^ (140 mM KCl, 5.4 mM NaCl) and low K^+^/high Na^+^ (5.4 mM KCl, 140 mM NaCl) buffer supplemented with 2 mM CaCl_2_. They observed an increase in the cytosolic calcium concentration when parasites were transferred from high K^+^ to low K^+^ buffer, but no difference in the cytosolic calcium concentration was found in the opposite condition, i.e., low K^+^ to high K^+^ for wild-type parasites [35].

The activation of PfSR25 by shifts in [K^+^] increases cytosolic calcium in *P. falciparum*. The increase in Ca^2+^ is blocked by the inhibition of phospholipase C (PLC) or depletion of internal Ca^2+^ pools of the parasite by thapsigargin. However, PfSR25 knockout parasites did not show an increase in cytoplasmic Ca^2+^ when subjected to changes in [K^+^] from 140 mM (intracellular) to 5.4 mM (extracellular) [35]. The authors concluded that PfSR25 is a K^+^ sensor capable of modulating Ca^2+^ signaling in *P. falciparum*, resulting in consequent PLC activation and increased [Ca^2+^] cyt. This response persists in the absence of free extracellular Ca^2+^ and cannot be elicited by other ions such as Na^+^, Mg^2+^, or Ca^2+^ [35].

Interestingly, the authors found that the knockout for the PfSR25 protein is more sensitive to oxidative stress promoted by sodium nitroprusside (SNP), a nitric oxide donor. When parasites were treated with SNP, a significant decrease in parasitemia was observed, and the knockout (PfSR25^-^) parasites showed a 72% increase in metacaspase gene expression after exposure to SNP 0.5 mM for 3 h compared to wild-type (3D7) parasites. The wild-type parasites also showed an improved ability to survive under amino acid deprivation conditions with the lack of Albumax as supplementation to media compared to the knockout parasites [35]. This data set highlights the involvement of PfSR25 in the other mechanisms during the erythrocyte cycle.

In a recent paper, Santos and colleagues reported that PfSR25 knockout parasites show increased susceptibility to synthetic compounds derived from 1H- and 2H-1,2,3-triazole [36]. The authors identified 31 compounds with potential antimalarial activity at concentrations in the micromolar (µM) order; then, using the PfSR25^−^ strain showed that compounds with antimalarial activity against the 3D7 strain (wild-type) showed reduced IC_50_ values when tested against the knockout strain [36]. In a further recent study, Santos and colleagues reported that PfSR25^−^ showed increased susceptibility to the antimalarial drugs lumefantrine and piperaquine, which target hemozoin metabolism [37]. The authors tested different antimalarial drugs used for treating malaria with varying mechanisms of action on the 3D7 and PfSR25^−^ strains. Among them are atovaquone (mitochondria), dihydroartemisinin and pyrimethamine (cytosol), chloroquine, mefloquine, lumefantrine, and piperaquine (digestive vacuole). As a result, they observed that the knockout parasites were more sensitive to the antimalarial activity of piperaquine and lumefantrine, presenting 44.38% and 58.12% lower IC_50_ values in the PfSR25^−^ strain when compared to the 3D7 strain [37].

In the erythrocytic cycle, malaria parasites digest hemoglobin within the digestive vacuole to obtain amino acids, releasing the toxic product heme from hemoglobin. During detoxification, the heme is converted into an insoluble crystal called hemozoin by the heme detoxification protein [38]. This detoxification process is essential for the parasite’s survival and is, thus, the target of some antimalarial drugs. Interestingly, the knockout strain (PfSR25^−^) shows greater susceptibility to some of these compounds, such as chloroquine, piperaquine, and lumefantrine [35,37]. These compounds can reduce PfSR25 expression in the ring phase [37,39]. In an assay, Moraes and colleagues treated PfSR25^−^ and wild-type 3D7 trophozoites (32–34 h post-infection) with piperaquine (10 μM) for 2 h and measured hemozoin formation, noting that PfSR25^−^ parasites were more susceptible to treatment. After 2 h, the knockout inhibited hemozoin size formation by approximately 69.9 ± 2.1% compared to the wild-type parasites [35]. Thus, besides acting in detecting the K^+^ shift, PfSR25 can play other functions related to parasite survival.

Signaling triggered by K^+^ shift is one of the mechanisms involved in the invasion of new cells. Moreover, other molecular factors act in this complex event, such as the activation of signaling pathways and specific proteins during the mechanical steps of invasion [40,41,42,43,44]. In addition to the involvement of PfSR25 in the detection of K^+^ shift, the data found with the study of antimalarial compounds in this strain demonstrate a relationship between the action of antimalarial drugs and this GPCR-like receptor. Therefore, PfSR25 is a promising candidate for studies of the development of new antimalarial.

## 5. Other Apicomplexans Encode Proteins Homologous to GPCR-like PfSR25

**Figure 2:**
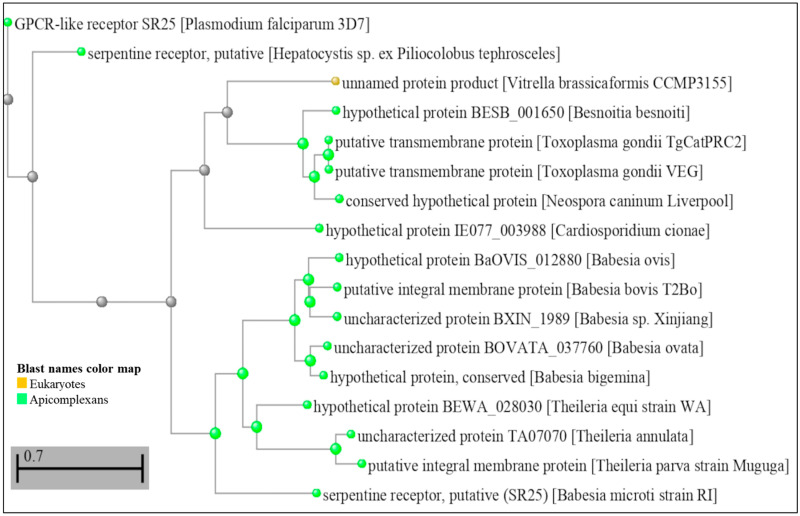
Dendrogram showing amino acid sequence comparison of PfSR25 (PF3D7_0713400) with other sequences from the US National Library of Medicine database—National Center for Biotechnology Information (NCBI—https://blast.ncbi.nlm.nih.gov/Blast.cgi accessed on 6 February 2023). A search was performed on the BLAST platform that resulted in significant alignment sequences; these data were later used to build a distance tree from the results performed on the platform itself [45,46]. (Scale bar 0.7 amino acid substitutions).

In *Toxoplasma gondii*, a similar mechanism for sensing [K^+^] has also been described, where the decrease of [K^+^] from intracellular to extracellular levels in the parasitized host cell results in a PLC-dependent increase of cytoplasmic Ca^2+^ [14]. According to the study, this increase in Ca^2+^ triggers a signal to activate the parasite egress machinery [14]. It is important to note that PfSR25, as well as having a conserved structure in *Plasmodium* spp., also appears to have homologs in other parasites. A BLASTP (protein–protein BLAST) search using PF3D7_0713400 as an input demonstrates that other species of apicomplexan parasites, such as *Hepatocystis* sp., *Besnoitia besnoiti*, *Toxoplasma gondii*, *Neospora caninum Liverpool*, *Cardiosporidium cinoae*, *Babesia* sp., and *Theileria* sp., as well as the unicellular algae *Vitrella brassicaformis* (belonging to the eukaryotic supergroup alveolata), showed sequences that produced significant alignments with PfSR25 and may represent true functional homologs in these other organisms (Figure 2).

These findings lead us to propose the hypothesis that parasite detection of [K^+^] shifts in the ionic environment may be conserved amongst different species of apicomplexan parasites. Active invasion of host cells is a characteristic of the apicomplexan, and it is tempting to suggest that this process may be regulated by sensing K^+^ shifts. In *P. falciparum*, this perception of K^+^ shift may be one of the mechanisms that ensure that the parasite remains in the host cell during intracellular replication and later becomes active; the increase in Ca^2+^cyt may be associated with the requirement for parasite activation of CDPKs (calcium-dependent kinases) in the release of microneme proteins essential for infection of new erythrocytes. At this time of egress/invasion, K^+^ displacement occurs, which promotes an intracellular Ca^2+^ increase by PLC action, which is sensed by the GPCR-like PfSR25.

## 6. Calcium Acts as the Second Messenger Resulting from the Signaling Triggered by [K^+^] Shift

Calcium is an essential ion for apicomplexan parasites, as it is involved in critical biological processes during the parasite life cycle. Classical mechanisms have been described for the uptake and release of calcium, which acts as a second messenger in signaling pathways. Some of these mechanisms are essential at different stages of the parasite life cycle [38,47,48]. Several processes, such as egress, invasion, motility, protein secretion, and cell cycle regulation, are controlled by Ca^2+^ signaling in the parasites [49,50]. The invasion of new red blood cells by the parasite is influenced by the extracellular ATP levels, which trigger an increase of Ca^2+^cyt in the parasite. Levano-Garcia and collaborators reported that the addition of ATP leads to an increase in Ca^2+^ in trophozoites and segmented schizonts. The presence of purinergic inhibitors KN62 and Ip5I was able to block the release of Ca^2+^ when ATP was added. In addition, it promoted a drastic reduction in the infection of new red blood cells [51].

It is worth emphasizing that *Plasmodium* can create a high Ca^2+^ microenvironment during its intraerythrocytic cycle [52]. Efforts by our group and others reported the host hormone, melatonin, as active in the synchronization of *P. falciparum* (in vitro) and *P. chabaudi* (in vivo) [52]. We also showed that melatonin and its precursors could mobilize Ca^2+^ from intracellular storage [52,53]. The signaling acting in the melatonin pathway involves the activation of the PLC-IP3 pathway that leads to the cytosolic increase of calcium and cAMP. Recently, Alves et al., 2022, identified PfMDR1 as a promising IP3 receptor candidate in *P. falciparum*, combining an IP3 affinity column chromatography with further bioinformatics meta-analysis. The analyses show that PfMDR1 is a potential target for binding IP3 [54]. Thus, we show that Ca^2+^ acts as a second messenger in several signaling pathways activated at different stages of development, essential for cycle progression and parasite survival [55].

Calcium-dependent protein kinases (CDPKs) play significant roles in the Ca^2+^ signaling pathways in parasites. Through crystallography, it was shown that CDPKS from *T. gondii* and *Cryptosporidium parvum*, in their self-inhibited forms (not bound to Ca^2+^), resembles a calmodulin protein with a long helix at the N-terminus that inhibits the protein’s kinase domain [56]. Lourido et al. (2010) used a knockdown of *T. gondii*, CDPK1, to demonstrate the essentiality of this kinase in the signaling that leads to the secretion of micronemes [57]. CDPK1 is a Ca^2+^-dependent kinase that is conserved among apicomplexan parasites. Its conditional deletion showed that CDPK1 controls Ca^2+^-dependent secretion from organelles called micronemes. As a result, the knockdown parasite was impaired in terms of motility, invasion, and egress from host cells. That is, in the stages where the release of the microneme is essential for the continuity of the parasite’s life cycle [57]. TgCDPK1 inhibitors (1NM-PP1) block microneme secretion and motility, promoting a blockade in invasion and parasitic development [58].

As it occurs in *T. gondii*, CDPK1 is also involved in the motility and invasion of new cells in *P. falciparum*. Green and collaborators showed that this Ca^2+^-dependent kinase is responsible for the phosphorylation of proteins MTIP (myosin A tail domain interaction protein) and GAP45 (glideosome-associated protein 45), both components of the motor complex that generates the necessary force to invade host cells [59]. Another relevant kinase in *P. falciparum* is PKB. This is an enzyme similar to protein kinase B, whose regulation is controlled by calmodulin, which in a Ca^2+^-dependent manner, associates with the N-terminal region of PfPKB and regulates activity [60]. The study of the Ca^2+^/Calmodulin-PfPKB signaling pathway showed that it might be necessary for erythrocyte invasion. Through the development of an inhibitory peptide for PfPKB, a drastic reduction in the ability of the parasite to invade new erythrocytes has been shown. This same result was also obtained when inhibitors of the activators of this pathway, calmodulin, and PLC, were used [61]. Thus, Ca^2+^-dependent kinases CDPK1 and PKB are highlighted as critical mediators of the signaling that leads to the secretion of micronemes, motility, and invasion in the parasite. Therefore, this set of data points to a likely conserved mechanism among parasites.

Exposure of *P. falciparum* merozoites to a low [K^+^] (5 mM), similar to that present in the bloodstream, induces the activation of phospholipase C, resulting in increased intracellular Ca^2+^ and activation of erythrocyte invasion mechanisms. Moraes et al. (2017) demonstrated that changing the KCl concentration from 140 mM to 5.4 mM induces an increase in cytosolic Ca^2+^ concentration in *P. falciparum* trophozoites, even in the absence of extracellular Ca^2+^ [35]. In *T. gondii*, a mechanism similar to the one mentioned above has also been described [14]. However, the GPCR-like 25 of *P. falciparum* presenting a representative homologous protein in the genome of *T. gondii*, the receptor responsible for the uptake of K^+^ levels and subsequent release of Ca^2+^cyt, was not characterized. In any case, it is vital to highlight the fundamental role of Ca^2+^ at the time of the invasion of new host cells, not only in *P. falciparum* and *T. gondii*, since studies show that in *Trypanosoma cruzi* elevations in Ca^2+^cyt levels are also present at the time of the invasion of new cells [62]. Another study showed that Ca^2+^ signaling during the invasion, as well as an increase in intracellular calcium levels, are linked to the virulence of *Leishmania mexicana amazonensis* [63].

There are still significant gaps to be filled in the events behind the parasite–host cell interaction processes (egress and invasion). However, Ca^2+^ is shown to be a ubiquitous intracellular messenger in different parasites. Although there are still many unanswered questions on this subject, identifying the SR25 protein in *P. falciparum* sheds light on new mechanisms by which parasites can capture information from the surrounding microenvironment and prepare themselves to initiate further steps in their life cycle.

## 7. Conclusions

In this present contribution, we review the importance of K^+^ ions in intracellular parasites, focusing on the human malaria parasite *P. falciparum* and the related parasite *Toxoplasma gondii*. Alterations in the concentration of this ion can be sensed by the GPCR-like PfSR25 and transduced to the parasite cytosol through Ca^2+^, a ubiquitous second messenger. This signaling performs different functions depending on the stage of the parasite’s development. However, it proves to be extremely important for the hepatic and intraerythrocytic stages of *Plasmodium* and other parasites. The [K^+^] detection mechanism is slowly unveiled and still has gaps to explore. Complete knowledge of this mechanism will contribute to our understanding of parasite cell biology and may also lead to new targets for the development of antimalarial drugs.
